# Hazelnut Yield Estimation: A Vision-Based Approach for Automated Counting of Hazelnut Female Flowers

**DOI:** 10.3390/s25103212

**Published:** 2025-05-20

**Authors:** Nicola Giulietti, Sergio Tombesi, Michele Bedodi, Carol Sergenti, Marco Carnevale, Hermes Giberti

**Affiliations:** 1 Dipartimento di Ingegneria Industriale e dell’Informazione, Università di Pavia, Via Adolfo Ferrata 5, 27100 Pavia, Italy; carol.sergenti@unipv.it (C.S.); marco.carnevale@unipv.it (M.C.); hermes.giberti@unipv.it (H.G.); 2 Department of Sustainable Crop Production, Università Cattolica del Sacro Cuore, 29122 Piacenza, Italy; sergio.tombesi@unicatt.it; 3 Department of Mechanical Engineering, Politecnico di Milano, Via La Masa 1, 20156 Milan, Italy; michele.bedodi@mail.polimi.it

**Keywords:** vision-based measurement system, female flower counting, hazelnut tree, *Corylus avellana*, image tiling

## Abstract

Accurate estimation of hazelnut yield is crucial for optimizing resource management and harvest planning. Although the number of female flowers on a flowering plant is a reliable indicator of annual production, counting them remains difficult because of their extremely small size and inconspicuous shape and color. Currently, manual flower counting is the only available method, but it is time-consuming and prone to errors. In this study, a novel vision-based method for automatic flower counting specifically designed for hazelnut plants (*Corylus avellana*) exploiting a commercial high-resolution imaging system and an image-tiling strategy to enhance small-object detection is proposed. The method is designed to be fast and scalable, requiring less than 8 s per plant for processing, in contrast to 30–60 min typically required for manual counting by human operators. A dataset of 2000 labeled frames was used to train and evaluate multiple female hazelnut flower detection models. To improve the detection of small, low-contrast flowers, a modified YOLO11x architecture was introduced by adding a P2 layer, improving the preservation of fine-grained spatial information and resulting in a precision of 0.98 and a Mean Average Precision (mAP@50-95) of 0.89. The proposed method has been validated on images collected from hazelnut groves and compared with manual counting by four experienced operators in the field, demonstrating its ability to detect small, low-contrast flowers despite occlusions and varying lighting conditions. A regression-based bias correction was applied to compensate for systematic counting deviations, further improving accuracy and reducing the mean absolute percentage error to 27.44%, a value comparable to the variability observed in manual counting. The results indicate that the system can provide a scalable and efficient alternative to traditional female flower manual counting methods, offering an automated solution tailored to the unique challenges of hazelnut yield estimation.

## 1. Introduction

**Figure 1:**
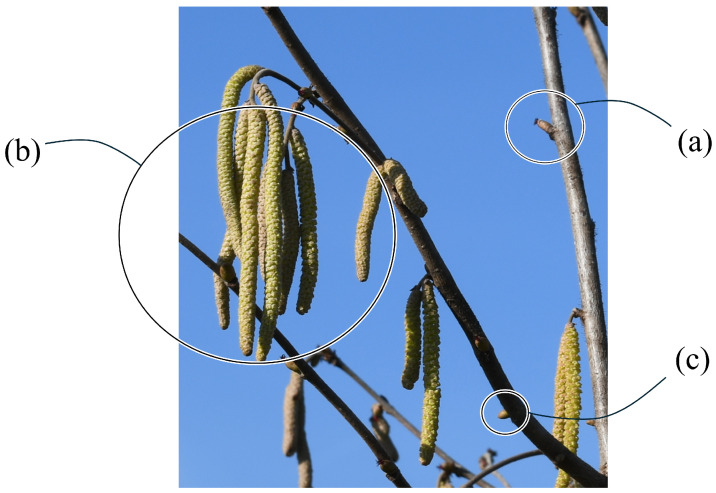
Distinction between female flower (a), male flower (b), and bud (c) in the hazel tree.

Agricultural yield forecasting is a key component of agribusiness supply chain optimization, directly influencing resource management, harvest planning, and market strategies. In recent years, with the advent of new technologies, data-driven approaches for predicting production trends have become increasingly important [1]. In fact, the annual production of various crops is highly dependent on many variables. While some factors, such as climatic events or diseases [2], cannot be controlled, predictive models have been developed over the years for others, such as the seasonality of plant growth and the number of inflorescences produced [3,4]. Filbert (*Corylus avellana*) cultivation is one of the most important tree nut crops, accounting for about 1.08 million hectares worldwide (FAOSTAT, 2023). Filbert is a monoecious diclinous species, meaning that reproductive structures are borne separately in female flowers, named glomerules, and male flowers, named catkins. Early estimation of annual yield is closely linked to the number of female flowers on the plant, and it is particularly complex because of the size of female flowers, which are very similar to vegetative buds except for a wisp of red pistils a few millimeters long (Figure 1). Hazelnuts develop from pollinated female flowers and form clusters containing 1 to 15 or more nuts per cluster, depending on the cultivar. Occasionally, individual hazelnuts are enclosed in a single husk, which ripens and turns brown as the fruit matures. Although the number of nuts per cluster cannot be precisely predicted, it is largely dependent upon cultivars; although it can be affected by environmental factors such as seasonal conditions, management, and agricultural practices [5,6]. While seasonal conditions are not easily predictable, a count of female flowers can be performed instead, which can provide an early indication of final hazelnut production, especially in low-crop years.

Traditionally, counting female flowers has been conducted through manual observations, a highly subjective, time-consuming process limited to small sample sizes [7]. This is further complicated by the fact that the female flower of the hazel plant is easily confused with a bud and can be distinguished only by the presence of red pistils at the end [8,9]. This characteristic makes the flower particularly difficult to detect through the tree canopy, even if the branches are bare during the flowering season.

In recent years, the exploitation of technologies such as remote sensing [10], digital twins, and virtual reality has advanced the agricultural sector, especially in the field of crop management, enabling the development of advanced monitoring techniques, predictive growth models [11], and training methods [12]. Artificial intelligence techniques, including computer vision, have been increasingly used to monitor agricultural crops and, specifically, to count flowers. Estrada et al. presented a deep learning-based approach for automatic flower counting in densely populated peach orchard images. Comparing YOLO-based detection (i.e., YOLOv5, YOLOv7, YOLOv8) [13] with density map estimation, the results show that density map prediction outperforms object detection, achieving a Mean Absolute Error (MAE) of 39.13 [14]. Yi et al. propose Light-FC-YOLO, a lightweight deep learning model for detecting and counting high-density flowers on complex backgrounds. By integrating C2f-Ghost and SPPF-LSKA modules, the model improves feature extraction while maintaining efficiency, achieving an MAE of 4.53. Their work aims to balance MAE and computational efficiency; in fact, the model achieves 23.5 fps, making it suitable for implementation on edge devices and thus applicable to smart agriculture systems [15]. Wang et al. present GhP2-YOLO, a YOLOv8-based deep learning model for rapeseed flower detection and counting, which integrates Ghost modules and a P2 sensing head to improve small object detection. The model achieves an mAP50 of 95%, an mAP50-95 of 78.2%, and an F1-score of 0.880, outperforming the basic YOLO models in detection accuracy [16]. Tan et al. present a three-view cotton flower counting method that exploits multi-object tracking and RGB-D images to improve accuracy in large-scale cotton fields. The system integrates YOLOv8 for flower detection and Recurrent All-Pairs Field Transforms for optical flow-based tracking, employing a constrained hierarchical clustering algorithm to eliminate duplicate counts from multiple camera views. The YOLOv8x model achieved an mAP of 96.4%, while the mean absolute percentage error was 6.22% [17]. Lin et al. present a deep learning framework for lychee flower counting using YOLACT++ for instance segmentation and FlowerNet, a novel density map regression model based on multitask learning. The VGG16-based model achieved an MAE of 47.71 and an RMSE of 61.78, with an R^2^ of 0.81 compared to manual counting, demonstrating strong predictive accuracy. The focus is not on the algorithm’s computation time but on addressing challenges related to the high flower density in the images [18]. Rahim et al. present a Faster R-CNN-based approach for detecting and counting tomato flowers in greenhouse environments, accounting for occlusions and varying illumination conditions. Using ResNet50 as the backbone, the model achieves an accuracy of 96.02% and a recall of 93.09%, with an error range of −4 to +3 flowers per image compared to manual counting [19]. Li et al. present an improved YOLOv5-based method for automatic rapeseed inflorescence counting using UAV RGB imagery to enhance accuracy and efficiency. The model integrates a Convolutional Block Attention Module (CBAM) to improve feature extraction, achieving an mAP of 93.6%, an F1-score of 88.7, an R^2^ of 0.966, and an RMSE of 52.1, outperforming Faster R-CNN, YOLOv4, and TasselNetV2+. This approach enables precise and rapid inflorescence quantification, demonstrating its potential for high-throughput plant phenotyping and yield prediction [20]. Yu et al. present A-pruning, a lightweight YOLOv5-based deep learning model for real-time pineapple flower detection and counting, utilizing filter pruning and an adaptive batch normalization evaluation mechanism to optimize performance. The resulting YOLOv5-E model achieves 71.7% mAP, with a model size of 3.8 MB and 1.7 M parameters, running at 178 FPS, almost twice the speed of the original YOLOv5, making it highly efficient for mobile and embedded agricultural applications [21].

### 1.1. Motivation and Novel Work

As shown in Table 1, the recent literature focuses heavily on practicality and versatility.

In all cases, simple RGB cameras are used to enable the application of flower counting models on portable or edge devices and to facilitate the use of simple artificial intelligence models, such as the YOLO models. Moreover, a trend emerges in which specific models are trained for each plant type. For filbert, however, the task is more complex. To estimate future crop productivity based on the count of female flowers, a reliable, practical, and fast methodology must be developed. Ideally, this method should require only a few images per plant, allowing its application across an entire crop or a large portion of it. Additionally, it should be economical, versatile, and usable by non-expert operators. Existing vision-based flower counting methods have mainly focused on crops with larger, high-contrast flowers, which are easier to detect using standard object detection frameworks. In contrast, female hazelnut flowers present unique detection challenges. The main challenge in counting female hazelnut flowers is their small size, making them almost undetectable to the naked eye except at close range. Moreover, their color, ranging from light to dark red, blends easily with the brownish tones of adjacent branches. Even with high-resolution imaging systems, each individual flower occupies only a few pixels in the image when capturing the entire plant in a single shot. These factors necessitate the use of specialized strategies that are not required in cases where inflorescences are larger and more conspicuous. To address this, an image-tiling strategy approach is introduced in this study to detect extremely small flowers within high-resolution images. The method involves acquiring a single high-resolution image per side of the plant (i.e., two sides), which is then decomposed into sub-images. After appropriate training, each sub-image is processed using a modified YOLO-based object detection model, in which a P2 layer was added to improve the preservation of fine-grained spatial information and enhance the detection of small, low-contrast flowers. Finally, a regression-based bias correction is performed to address the inherent limitations of the method. Given its simplicity and speed, the method relies solely on acquiring two high-resolution images. This introduces uncertainty in the estimation of the total number of flowers, as it is not guaranteed that numerous flowers are included or counted twice in both frames. Different object detection model architectures and hyper-parameter sets are compared. The total number of detected flowers is then analyzed and compared with manual counts performed by experienced field operators. In summary, this study introduces the following contributions:

- A robust methodology that combines state-of-the-art object detection models with a regression-based bias correction, requiring only two high-resolution images per plant, enabling practical large-scale application even by non-expert operators;
- An image-tiling strategy combined with a YOLO-based model to detect extremely small, low-contrast flowers;
- A custom architecture of the YOLO11x model with the addition of a P2 layer to improve the detection of small objects by enhancing fine-grained spatial resolution;
- A comparative analysis of different object detection models to evaluate their effectiveness for this specific task;
- An evaluation of the proposed method in a real hazelnut field compared with manual counts by experienced operators in the field.

### 1.2. Organization of the Paper

This paper is structured as follows. Section 2 presents the proposed method for counting female hazelnut flowers, illustrating the data acquisition process, image processing pipeline, and training of the object detection model. Section 3 reports experimental results, including evaluation of different object detection models, performance comparison, and field validation against manual counting. Finally, Section 4 summarizes the results obtained, the limitations of the method, and future steps.

## 2. Materials and Methods

**Figure 2:**
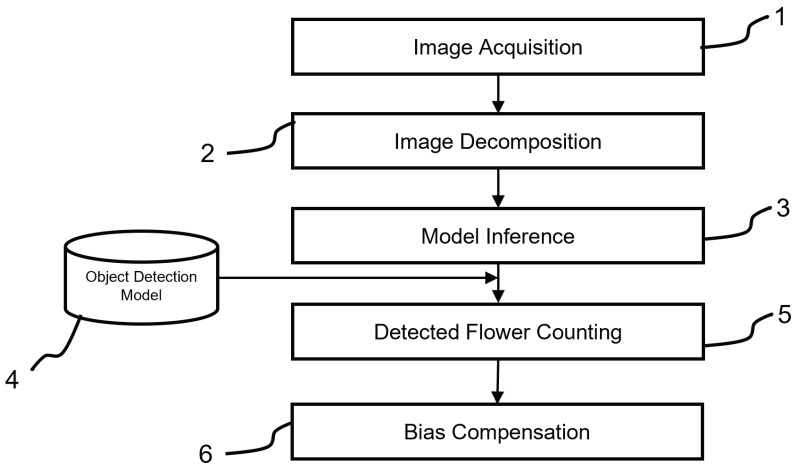
Visual workflow of the proposed flower counting method. Acquisition of high-resolution RGB images from both sides of the hazelnut plant (1). Division of the images into sub-images (2). Application of a YOLO-based detection model to each tile (3). Identification of female flowers within tiles (4). Aggregation of results (5) and correction using a linear regression model to estimate the total number of female flowers per plant (6).

The method for estimating the number of female flowers on a hazelnut plant is schematized in Figure 2.

**Figure 3:**
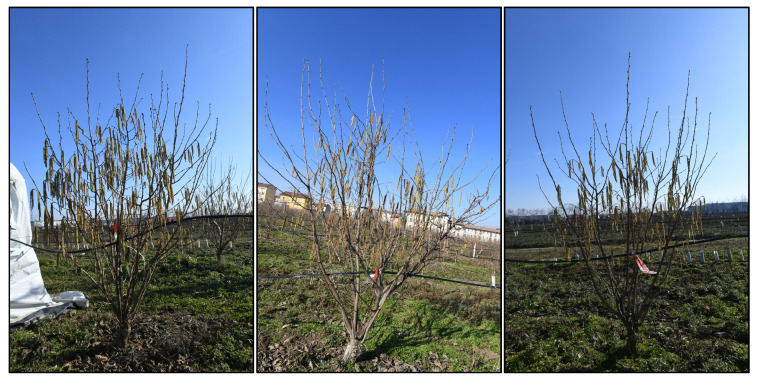
Example of acquired high-resolution images of the hazelnut tree.

**Figure 4:**
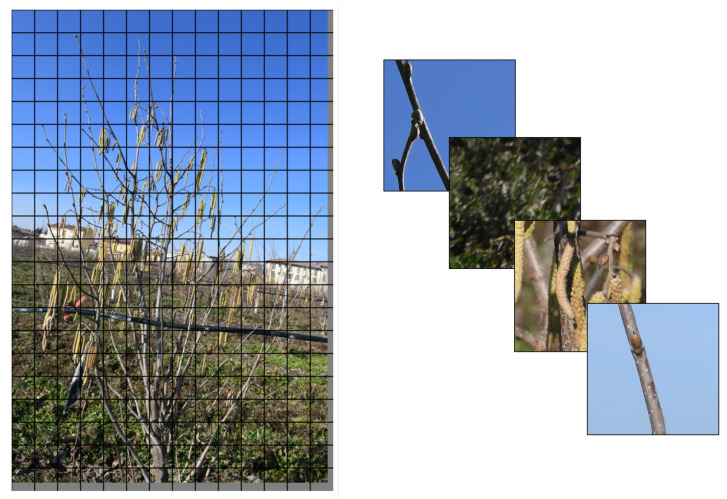
Schematic representation of the image-tiling-based method. The high-resolution image of the entire tree is divided into smaller square sub-images to facilitate object detection. Each sub-image is processed individually, allowing for improved identification of small objects, such as female hazelnut flowers.

**Figure 5:**
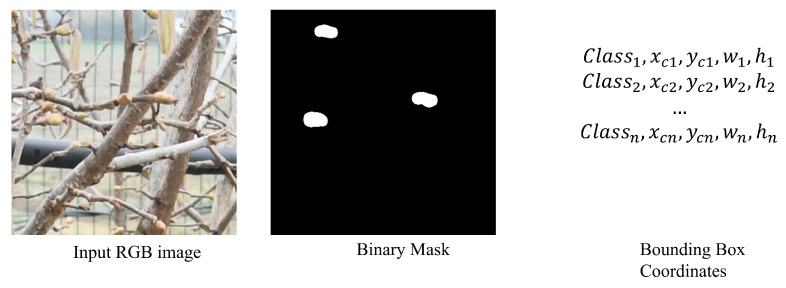
Example of an input RGB image and its corresponding binary mask, highlighting the detected regions of interest. For each highlighted contour, the normalized coordinates of the minimal enclosing rectangle are provided.

As introduced in Section 1.1, the method is designed for field application by unskilled operators using commercial hardware. The process begins with the operator acquiring two images of the plant from opposite sides (Figure 2—1) using a high-resolution RGB camera. The images are divided into square sub-images of reduced size, also called image tiling (Figure 2—2), and fed into a previously trained object detection model (Figure 2—3), which identifies the location of female flowers within each sub-image (Figure 2—4). The total number of flowers is obtained by summing all detected flowers across the sub-images (Figure 2—5). An example of a captured image is shown in Figure 3, where the hazelnut plant is framed in its entirety. It can be seen that the male flowers are clearly visible, while the female flowers are difficult to distinguish.

The approach just described involves several simplifications. Notably, there is no guarantee that all female flowers on the plant will be captured, as some may be obscured by branches, catkins, or other elements. Additionally, the method introduces the risk of double-counting flowers visible from both sides. However, these simplifications are required to ensure the practicality and speed of the method, which is designed for large-scale application across extensive land areas and numerous plants. Finally, the results compared with counts performed by experienced operators are used to develop a bias correction model to account for these simplifications and refine the final measurement (Figure 2—5).

### Object Detection Model Training

Since no object detection model for female hazelnut flowers is available in the literature, a new model must be trained from scratch.

The first step involves acquiring a large dataset of high-resolution images that includes as many flowering hazelnut trees as possible, captured under diverse environmental conditions (e.g., sunny, cloudy, foggy). It is essential to plan the acquisition during the appropriate phenological phase to ensure the presence of visible female flowers. A resolution of at least 24 megapixels is recommended to guarantee sufficient pixel density for detecting small floral structures even when framing the entire plant. One critical aspect is the depth of field, which, in this case, is influenced by the lens’s focal length, aperture, and focus distance. To reduce geometric distortion while preserving sufficient depth of field, lenses with an effective focal length of 24 mm (full-frame equivalent) should be used. Shorter focal lengths are not recommended due to increased barrel distortion. The use of high-quality lenses is essential to maintain fine details throughout the frame. The shooting distance should be minimized, while still allowing the full plant to be captured in the frame. The camera should be positioned at the center height of the plant to ensure uniform focus distribution. Regarding aperture, in order to maximize depth of field without inducing diffraction-related degradation, the aperture should be stopped down as much as possible within the limits recommended by the lens’s Modulation Transfer Function chart. The acquired high-resolution images of the entire tree are divided into smaller square sub-images (image tiling), adding padding to the edges if necessary (Figure 4). Padding is applied only when the image dimensions are not divisible by 400, in order to allow complete tiling without leaving unprocessed regions. Tiling-based methods have proven particularly effective for identifying small objects in high-resolution images [22,23].

Images of the entire tree, collected for training and decomposed into sub-images, are processed by an experienced operator who examines the images, identifies female flowers, and annotates them using a graphical interface developed specifically for this task in Python.

This process generates binary masks in which pixels have a value of 1 (white) if they correspond to a female flower and 0 (black) otherwise. The binary masks are finally converted into a bounding box (BB) list. To achieve this, the contours of the segmented regions are identified and then the minimum rectangle enclosing each contour is calculated, resulting in a list of BB coordinates. Each BB is represented by its class (in this case, it will be just class 0 for female hazelnut flower), and by the coordinates (normalized between 0 and 1 with respect to the mask size) of the center $\left(x_{c},y_{c}\right)$ and width, height (w,h) of the BB (Figure 5).

Each sub-image corresponds to a mask, which in turn corresponds to a list of normalized BB coordinates with their associated class. The training dataset, consisting of RGB sub-images and their corresponding normalized BB coordinates, is used for training object detection models. Given its widespread use for similar tasks (Section 1.1), the YOLO model family is chosen as the basis for training. The Mean Average Precision (mAP) metric, computed over Intersection over Union (IoU) thresholds ranging from 50% to 95% (mAP@.50-95), is chosen as the objective function to be optimized. The IoU measures the overlap between the predicted BB and the ground-truth BB and is defined as Equation (1), where ${\mathrm{BB}}_{\mathrm{pred}}$ represents the predicted BB, while ${\mathrm{BB}}_{\mathrm{gt}}$ represents the ground-truth BB. The IoU value ranges from 0 (no overlap) to 1 (perfect overlap). A detection is considered correct, or a True Positive (TP), if its IoU with the ground-truth is greater than or equal to a predefined threshold, typically varying from 0.50 to 0.95. If the predicted BB does not sufficiently overlap with any ground-truth object, it is considered a False Positive (FP). If a ground-truth object is not detected by the model, it is counted as a False Negative (FN).

(1) $$\mathrm{IoU}=\frac{|{\mathrm{BB}}_{\mathrm{pred}}\cap {\mathrm{BB}}_{\mathrm{gt}}|}{|{\mathrm{BB}}_{\mathrm{pred}}\cup {\mathrm{BB}}_{\mathrm{gt}}|}$$

Also, precision and recall are common evaluation metrics for object detection models. Precision (Equation (2)) quantifies the proportion of correct detections among all model predictions, while recall (Equation (3)) measures the proportion of actual objects in the scene that have been correctly detected.

(2) $$\mathrm{Precision}=\frac{\mathrm{TP}}{\mathrm{TP}+\mathrm{FP}}$$

(3) $$\mathrm{Recall}=\frac{\mathrm{TP}}{\mathrm{TP}+\mathrm{FN}}.$$

The Average Precision (AP) quantifies the area under the precision–recall curve and provides a single scalar value describing the model’s detection accuracy at a given IoU threshold. It is computed as Equation (4) where *M* is the number of recall levels considered, $P(R_{k})$ is the interpolated precision at recall level $R_{k}$, and $\Delta R_{k}$ is the difference between consecutive recall levels.

(4) $$AP=\sum_{k=1}^{M}P\left(R_{k}\right)\Delta R_{k}$$

Finally, the mAP@50-95 is obtained by computing AP at different IoU thresholds, ranging from 0.50 to 0.95 in steps of 0.05, and averaging the results across all classes (Equation (5)). Here, *N* represents the total number of object classes (i.e., one class in this case), $AP_{i}\left({\mathrm{IoU}}_{j}\right)$ is the Average Precision for class *i* at IoU threshold ${\mathrm{IoU}}_{j}$, and ${\mathrm{IoU}}_{j}$ varies from 0.50 to 0.95 in increments of 0.05. Unlike mAP@50, which evaluates performance at a single IoU threshold (0.50), mAP@50-95 provides a more exhaustive measure of the model’s ability to detect objects under varying degrees of strictness.

(5) $$\mathrm{mAP}\text{@}50\text{-}95=\frac{1}{N}\sum_{i=1}^{N}\left(\frac{1}{10}\sum_{j=1}^{10}AP_{i}\left({\mathrm{IoU}}_{j}\right)\right).$$

Training an object detection model involves the selection of several hyper-parameters. To optimize the training hyper-parameters of the model, a Bayesian optimization technique is applied, exploiting the Optuna Python library implementation [24]. This approach iteratively refines parameter selection based on previous evaluations, enabling a more efficient search compared to conventional methods such as random or grid search [25,26,27]. The objective function guiding the optimization is the maximization of mAP@.50-95, evaluated on the validation set (which is set as 20% of the training dataset). Training runs for a maximum of 1000 epochs, with an early stopping criterion that halts training if validation performance stagnates for 50 consecutive epochs. The learning rate varies between $10^{-3}$ and $10^{-6}$, while batch sizes range from 2 to 32. The optimizer is selected from a set of options, including SGD, Adam, AdamW, NAdam, RAdam, and RMSProp [28]. To ensure robust evaluation, a 3-fold cross-validation strategy is applied for each iteration.

## 3. Results and Discussion

### 3.1. Dataset

**Figure 6:**
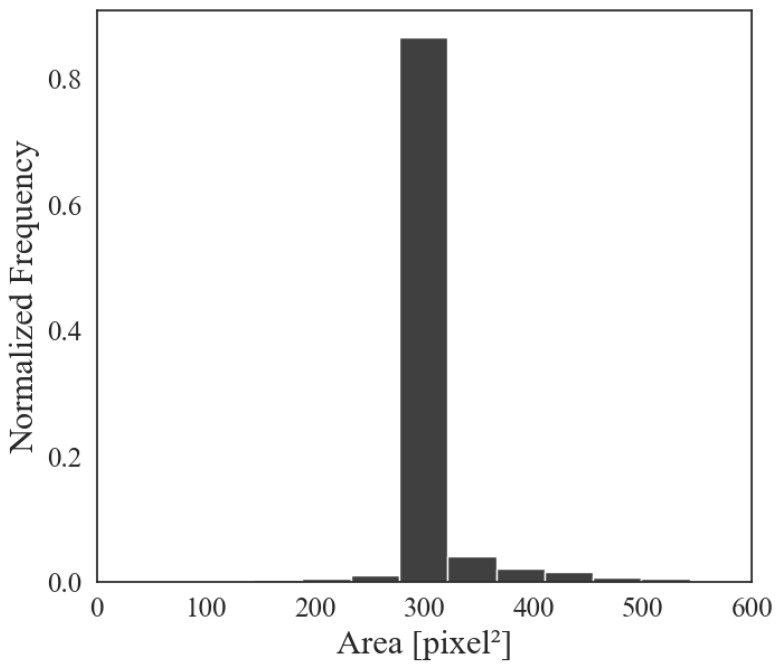
Histogram showing the distribution of manually segmented female inflorescence areas, with most values ranging between 280 and 320 pixel^2^.

In order to train the object detection model for automatic detection of female hazelnut flowers, 50 different high-resolution images were collected with a Nikon D850 SLR camera (Nikon Corporation, Tokyo, Japan) with a 8256×5504 pixel (i.e., 45.4 MP) CMOS sensor and Nikon AF-S 16–35 mm f/4 G ED VR lens (Nikon Corporation, Tokyo, Japan). The images depict the hazelnut plant in its entirety and were taken on different days under different weather conditions (e.g., sun, clouds, and fog). In each image, through the use of an interface developed in Python, the portions of the image comprising female flowers are selected. As shown in Figure 6, the average area of manually segmented inflorescence, in 83% of cases, is between 280 and 320 pixel^2^. This corresponds to a typical flower diameter of approximately 17–20 pixels. To ensure that multiple flowers are clearly detectable with sufficient spatial context, a tile size of 400×400 pixel was selected.

**Figure 7:**
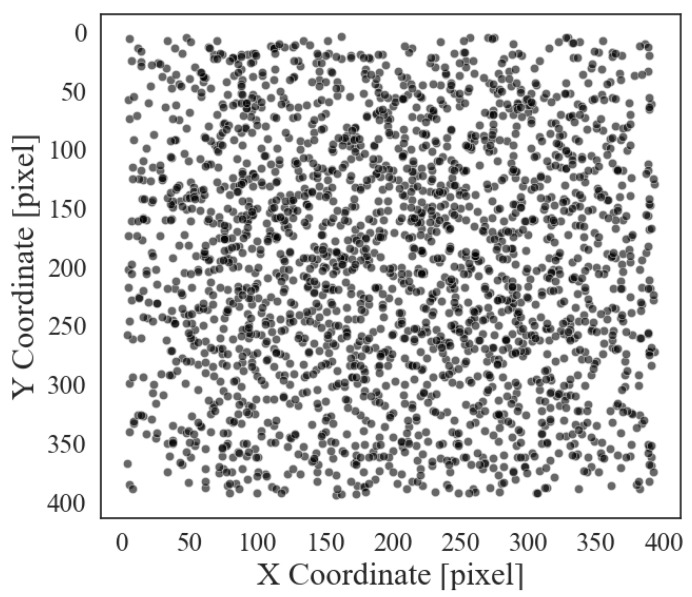
Spatial distribution of centroids of manually segmented areas at female flowers within sub-masks of size 400×400 pixels.

The 50 manually captured and segmented images are then divided into sub-images as explained in Section 2, resulting in a total of 14,700 images. In order to obtain a balanced dataset between the sub-images including female flowers and sub-images without female flowers, the dataset is randomly reduced to 2000 images, containing 2545 female flowers, 30% of which are completely free of female flowers. As shown in Figure 7, the centroids of the areas containing the flowers appear to be evenly distributed within the frames. RGB images and their respective masks are used for training the object detection model.

### 3.2. Model Training

The object detection model is trained using the collected dataset following the procedure described in Section 2. Multiple training runs are performed testing different models from the latest YOLO series: YOLOv8 [29], YOLOv9 [30], YOLOv10 [31], and YOLO11 [32]. Each version of YOLO differs in turn according to the number of parameters; the complete list is given in the Table 2. All models are evaluated, as heavier models with more trainable parameters generally achieve faster inference but do not always outperform lighter models in object detection accuracy [33]. All training procedures are conducted on an Ubuntu 22.04 system equipped with *NVIDIA RTX 4090* 24 GB GPU, *Intel Core i9-13900HX*, Python 3.10, PyTorch 2.3.1 and CUDA 12.1. For each training run, hyper-parameters are optimized (Section 2) to maximize mAP@.50-95 for each model. The optimization process is performed over 100 iterations. Results are reported in Table 2. However, YOLO11x obtains the highest mAP@50-95 (0.85) and a recall (0.95), suggesting its overall quality of detection. Although this results in a relatively higher inference time (8.52 ms) and model size (56 million parameters), it is still suitable for applications such as this one, where accuracy in object detection is the main driver.

**Figure 8:**
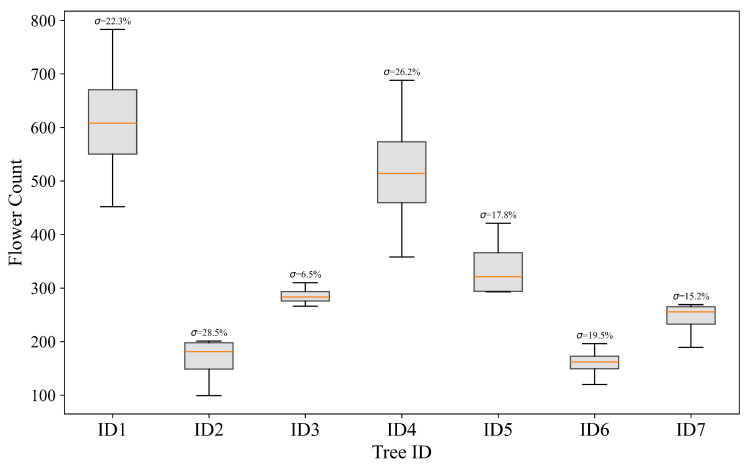
Box plot of manual counting of female flowers among the four operators for each hazelnut tree.

Based on the results obtained with the YOLO11x model (mAP@50-95 = 0.85; precision = 0.98; recall = 0.95), a modification to the architecture is introduced to improve the detection of small objects and early-stage visual features. A P2 detection head, already proposed for the YOLOv8 model in [16], was added to enable predictions at higher spatial resolution. The P2 head improves localization in scenarios involving fine details or small objects. This enhances the receptive capacity of shallow layers under challenging conditions [34,35]. The modified model, referred to as YOLO11x-P2, achieves an improved mAP@50-95 of 0.89, while precision (0.98) and recall (0.95) remain high. This reflects improved accuracy without loss in classification reliability. The added P2 head slightly increases computational cost; in fact, the inference time increases from 8.52 ms to 13.18 ms. Nevertheless, the model remains suitable for real-time inference on high-end embedded devices. The entire processing pipeline, from image tiling to flower detection, requires less than 8 s per plant (two sides), allowing rapid analysis on a large number of trees. This represents a substantial reduction in time compared to manual counting, which typically takes 30 to 60 min per plant and has significant variability among operators, as shown in Figure 8. The results demonstrate that adding a P2 head effectively enhances detection, particularly for small or occluded objects, with minimal impact on model inference time.

### 3.3. Field Model Testing

To field-test the model in a real use case, seven hazelnut plants were selected from a filbert cultivation. Four experienced operators manually counted the flowers on each plant, taking between 30 and 60 min per plant. Figure 8 shows box plots of the number of female hazelnut flowers counted on the seven plants by four independent operators. The analysis reveals significant variability among observations, with differences in both data dispersion and consistency among operators. Plants ID1 and ID4 show the greatest absolute variability, with standard deviations exceeding 130 units, while plant ID2 shows the highest relative variability, with a percentage standard deviation of 28.5%. The presence of extreme values in some plants suggests potential difficulties in manual counting due to environmental or subjective factors. This indicates that the widely used manual measurement method is inherently affected by significant uncertainty. Measurement uncertainty may arise from various factors. Specifically, the operator typically counts from top to bottom, but factors such as canopy width, fatigue, poor visibility of flowers, the temporal length of the count, or recounting previously counted flowers can lead to substantial errors.

**Figure 9:**
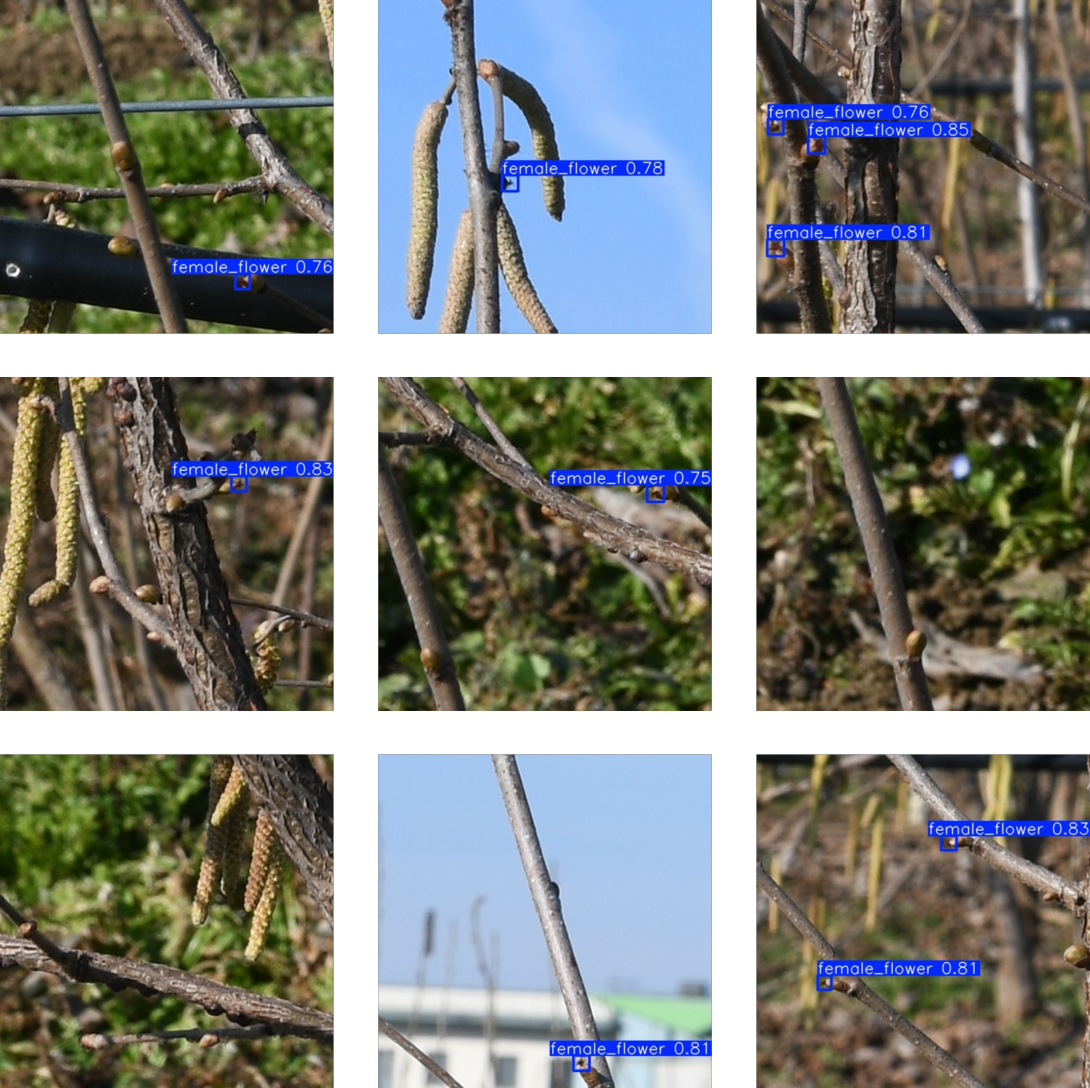
Examples of female hazelnut flower detection with the YOLOv11x-P2 model. The bounding boxes indicate the detected instances.

**Figure 10:**
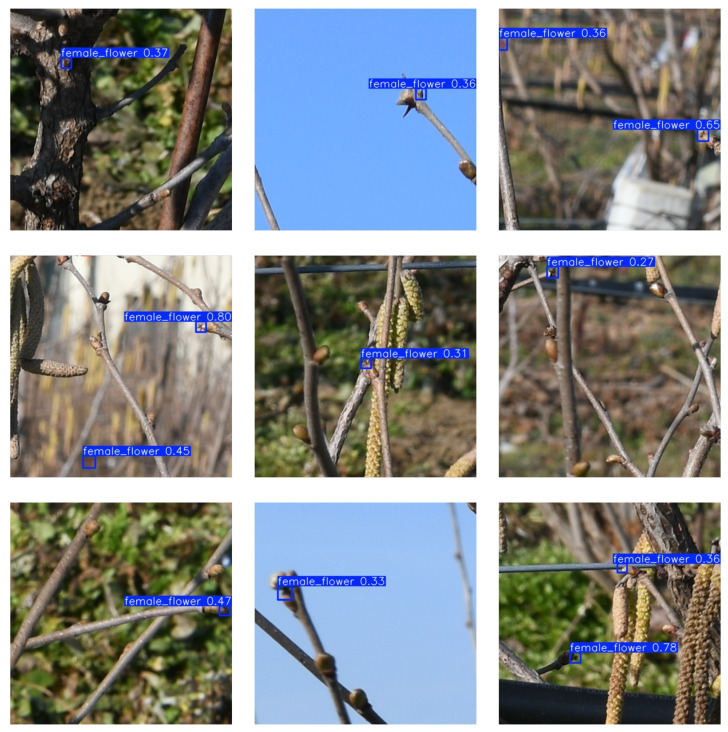
Examples of incorrect detections by the YOLOv11x-P2 model. These cases include False Positives, such as buds or branches mistakenly identified as flowers, often caused by visual noise, harsh shadows, or image blurring.

The approach described in Section 2 is applied to images of the seven hazelnut plants to obtain the total flower count per plant. After image tiling, each 400×400 sub-image is provided as input to the proposed YOLO11x-P2 object detection model, trained specifically for this task. The model outputs the positions of the female hazelnut flower within the image and assigns a confidence score, representing the estimated probability that a given detection corresponds to an object of the predicted class (Figure 9). All predictions with a confidence value below 0.5 are automatically discarded to reduce False Positives. Figure 10 presents nine examples where the female flower is misidentified as other plant elements with a confidence value below 0.5.

The ground truth is defined as the average number of female flowers counted by the four operators. As show in Table 3, although the YOLO11x-P2 model demonstrates high detection performance (mAP@50-95 = 0.89), raw prediction outputs tend to systematically underestimate the actual number of female flowers, with the exception of Tree ID 2. As shown in Section 2, this method introduces uncertainty primarily caused by factors such as partial occlusions, flowers located outside the field of view, and duplicate detections between the two image sides of the same plant.

**Figure 11:**
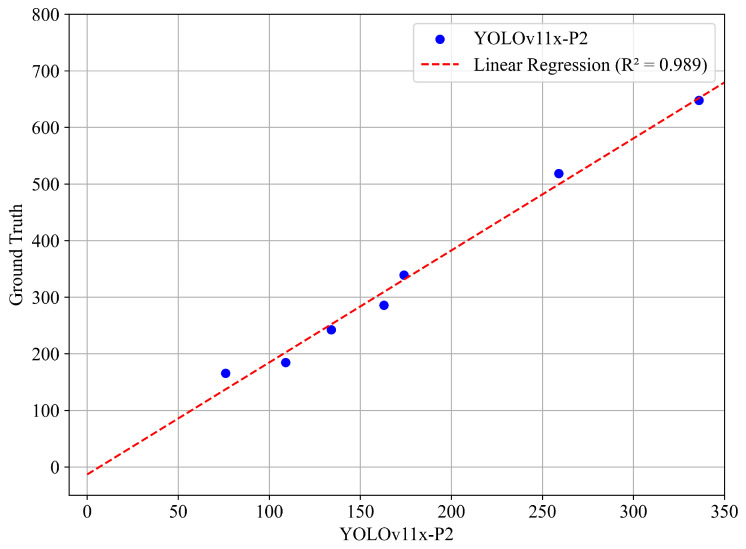
Scatter plot of the raw YOLO11x-P2 predictions versus ground truth values. The dashed red line indicates the linear regression fit y=1.980x+13.311 trained on a subset of the data, achieving an $R^{2}=0.989$.

The proposed method achieved an $R^{2}=0.989$. The resulting regression equation is y=1.980x+13.311 (Figure 11), with 95% confidence intervals ranging from 1.73 to 2.22 for the slope, and from –61.67 to 35.05 for the intercept. The raw forecasts obtained a mean absolute percent error (MAPE) of 37.29%, indicating a significant tendency to underestimate ground truth. In contrast, the corrected forecasts obtained by applying the interpolation function resulted in a significantly lower MAPE of 6.54%, with a standard deviation of 5.25% across the seven samples used for calibration, result entirely in line with the inter-operator variability observed previously (Figure 8). Similarly, the MAE drops from 141.75 to only 15.81 after applying linear interpolation.

Inference time analysis shows that the YOLO11x-P2 model, while offering the best accuracy (mAP@50-95 = 0.89), maintains an inference time of less than 13.18 ms per sub-image, making it suitable for field applications and integration on edge systems with limited computational capacity. The method takes a total of 3.9 s to calculate the number of female flowers on a single image (each image is composed of 294 sub-images), which can be up to one hour per tree.

## 4. Conclusions

In this study, a vision-based measurement system is developed for estimating the number of female flowers in hazelnut plants (*Corylus avellana*) using a commercial high-resolution imaging system and object detection models. The proposed approach is tested on field-collected images, demonstrating its ability to provide an automatic, rapid, and scalable alternative to manual flower counting. Among the evaluated models, the proposed YOLO11x-P2 achieved the best performance, with an $R^{2}$ of 0.989 and a MAPE of 6.54% after regression-based bias correction, a level of accuracy comparable to intra-operator variability in manual counting. The results indicate that the system can be applied in agricultural settings by being a practical tool for early yield estimation and allowing for an increase in the number of trees per hectare sampled for flower yield forecasting. Inference time analysis confirms that the trained model is computationally efficient (3.9 s per image), making it suitable for field use even with edge computing devices. Compared with manual counting, which can take up to an hour per plant and is subject to high variability among operators, the proposed system offers a time-saving advantage, requiring less than 8 s per plant, paving the way for large-scale use. Future work will focus on improving the robustness of the survey by increasing the available dataset, which is currently limited by the strong seasonality of the event, as the plants only flower for a few weeks of the year. The use of generative artificial intelligence techniques will then be explored in order to augment the dataset synthetically. Further validations will be conducted under different environmental conditions and hazel cultivars to test the methodology and verify its generalizability. These efforts will assess the generalizability of the method across orchards with different canopy architectures and cultivars, where flower visibility may vary significantly. Complex canopy structures can increase the risk of partial occlusion, while different lighting conditions—such as direct sunlight or shadow—may alter the visual features of flowers. Such variability may reduce detection reliability and affect the applicability of the regression model, which has been calibrated under specific acquisition conditions.

## Figures and Tables

**Table 1:** Comparison of deep learning-based flower counting methods. All percentages refer to the values reported in the cited works.

Reference	Base Model	Technique	Results	Vision System	Target Plant
Wang et al. (2024) [16]	YOLOv8m	P2 head, Ghost modules	Precision: 86.1% Recall: 84.4%	RGB camera	Rapeseed
Yi et al. (2024) [15]	YOLOv8s	Feature fusion	mAP@50: 87.0% Recall: 81.1%	RGB camera	Not specified
Yu et al. (2024) [21]	YOLOv5	Filter pruning, StrongSORT	mAP: 71.7% Recall: 72.0%	RGB camera (mobile)	Pineapple
Rahim et al. (2020) [19]	Faster R-CNN	Region-based CNN, thresholding	Precision: 96.02% Recall: 93.09%	RGB camera	Tomato
Lin et al. (2024) [18]	YOLACT++	Multitask learning	AP@50: 84.8%	RGB camera	Lychee
Tan et al. (2024) [17]	YOLOv8x	Deep optical flow	Precision: 96.4% $R^{2}$: 0.92	RGB-D	Cotton

**Table 2:** Comparison of YOLO model performances.

Model	mAP50_95	Precision	Recall	Inference Time	Params
	[-]	[-]	[-]	[ms]	[-]
yolo11x	0.85	0.98	0.95	8.52	56,874,931
yolo11l	0.79	0.96	0.93	4.62	25,311,251
yolo11m	0.80	0.96	0.96	3.94	20,053,779
yolo11s	0.82	0.95	0.94	2.02	9,428,179
yolo11n	0.73	0.94	0.91	1.23	2,590,035
yolov10x	0.78	0.96	0.92	8.95	31,656,806
yolov10b	0.85	0.97	0.94	4.73	20,452,566
yolov10l	0.84	0.97	0.96	5.83	25,766,870
yolov10m	0.83	0.96	0.94	3.76	16,485,286
yolov10s	0.84	0.96	0.95	2.40	8,067,126
yolov10n	0.76	0.94	0.91	1.32	2,707,430
yolov9e	0.81	0.95	0.94	10.98	58,145,683
yolov9c	0.81	0.95	0.95	5.18	25,530,003
yolov9m	0.80	0.96	0.94	4.18	20,159,043
yolov9s	0.69	0.92	0.91	2.19	7,287,795
yolov9t	0.79	0.94	0.92	1.55	2,005,603
yolov8x	0.85	0.95	0.97	9.32	68,153,571
yolov8l	0.85	0.96	0.92	5.86	43,630,611
yolov8m	0.81	0.96	0.92	3.59	25,856,899
yolov8s	0.79	0.94	0.93	1.59	11,135,987
yolov8n	0.79	0.94	0.95	0.98	3,011,043

**Table 3:** YOLO11x-P2 per-side predictions and their totals, compared to corrected ground truth and interpolated values.

Tree ID	Side	YOLO11x-P2 (Side)	YOLO11x-P2 (Total)	GroundTruth	YOLO11x-P2 (Interp.)
1	a	54	150	184.50	202.46
	b	96			
2	a	75	222	165.50	137.14
	b	147			
3	a	47	199	285.75	309.36
	b	152			
4	a	76	259	518.50	499.39
	b	183			
5	a	163	163	339.00	331.13
	b	0			
6	a	102	336	647.75	651.82
	b	157			
7	a	89	175	242.25	251.95
	b	85			
				MAE = 15.81	*R*^2^ = 0.989

## Data Availability

Dataset available on request from the authors.
